# Identification of Autoreactive B Cell Subpopulations in Peripheral Blood of Autoimmune Patients With Pemphigus Vulgaris

**DOI:** 10.3389/fimmu.2019.01375

**Published:** 2019-06-14

**Authors:** Robert Pollmann, Elias Walter, Thomas Schmidt, Jens Waschke, Michael Hertl, Christian Möbs, Rüdiger Eming

**Affiliations:** ^1^Department of Dermatology and Allergology, Philipps-Universität Marburg, Marburg, Germany; ^2^Institute of Anatomy and Cell Biology, Ludwig-Maximilians-Universität München, Munich, Germany

**Keywords:** autoimmunity, pemphigus vulgaris, desmoglein 3, B cells, flow cytometry

## Abstract

Pemphigus vulgaris (PV) is a rare blistering disease caused by IgG autoantibodies against the epidermal adhesion molecules desmoglein (Dsg)3 and Dsg1 providing a well-characterized paradigm of an antibody-mediated organ-specific autoimmune disease. In PV patients who have achieved clinical remission after B cell-depleting therapy, relapses often coincide with a reoccurrence of B cells and Dsg-specific autoantibodies. Here, we analyzed Dsg3-specific B cell subpopulations (i.e., total CD19^+^ B cells, CD19^+^CD27^−^B cells, CD19^+^CD27^+^ memory B cells, and CD19^+^CD27^hi^CD38^hi^ plasmablasts) in peripheral blood of both PV patients (*n* = 14) at different stages of disease and healthy individuals (*n* = 14) by flow cytometry using fluorescently labeled recombinant human Dsg3 protein. Applying this approach, Dsg3-specific B cells could be detected at low frequencies (0.11–0.53% of CD19^+^ B cells) and numbers of Dsg3-specific memory B cells were significantly increased in PV patients in clinical remission receiving minimal immunosuppressive therapy. Finally, we confirmed *in vitro* that Dsg3-reactive memory B cells were able to produce anti-Dsg3 IgG autoantibodies upon *ex vivo* activation. Thus, monitoring of Dsg3-specific B cells in PV is of particular interest to further characterize the immunopathogenesis of PV.

## Introduction

Pemphigus vulgaris (PV) is an autoimmune disease characterized by chronic and progressive erosive lesions of the mucosa, and blister formation at the suprabasilar layer of the skin caused by IgG autoantibodies (auto-ab) against adhesion molecules of the epidermis (1–3). The desmosomal cadherin desmoglein (Dsg)3 is the major autoantigen of PV with mucosal-dominant type while in patients with mucocutaneous PV, i.e., affecting mucosa and skin, auto-ab against Dsg1 are additionally found (4, 5). In the majority of PV patients, anti-Dsg IgG auto-ab correlate with disease activity (6, 7) and are therefore regularly used in routine diagnostics (8). The pathogenic relevance of auto-ab against Dsg3 has been shown in several *in vitro* and *in vivo* models by causing loss of keratinocyte cohesion (9–12), whereas a synergistic effect with other non-desmoglein autoantibodies is currently discussed (13, 14). Based on the well-described pathogenesis, the characterized autoantigens and the fact that Dsg-reactive IgG auto-ab are sufficient to cause blisters, PV is considered as a paradigm of an antibody-mediated organ-specific autoimmune disease. Moreover, PV serves as a model disease for the characterization of autoimmune mechanisms that finally lead to the generation of autoantigen-specific antibodies (15).

The B cell-depleting monoclonal anti-CD20 antibody rituximab leads to a marked decrease of Dsg3 auto-ab-titers paralleled by a fast clinical remission in the majority of PV patients (16–18), underlining the crucial role of continuous auto-ab production in PV by Dsg3-specific B cells, plasmablasts, and plasma cells.

Although the majority of patients achieve clinical remission after rituximab treatment, clinical relapses occur frequently in PV patients on long-term follow-up with reoccurrence of B cells and Dsg3 auto-ab in peripheral blood (19). This data suggests that Dsg3-specific B cells reappear at a certain time point during remission providing the base for a potential disease relapse. However, whether clinical relapses result from either Dsg3-specific B cells that have not been completely depleted by therapy or by *de novo* generated autoreactive B cells has not yet been fully elucidated. Genetic characterization of anti-Dsg3-IgG produced by B cells from PV patients indicates that patients with recurrent disease maintain a limited set of autoreactive Dsg3-specific B cell clones that persist over time (20). In contrast, using proteomic analysis of serum auto-ab, a recent study revealed a much more polyclonal and diverse pool of IgG auto-ab in PV (21).

To further examine the persistence of autoreactive peripheral blood B cells in pemphigus, we sought to characterize Dsg3-specific B cell subpopulations (i.e., mature naïve, memory, and plasmablasts) in PV patients at different stages of disease utilizing fluorescently labeled recombinant human Dsg3 (Dsg3-AF647) like it has been previously demonstrated for other antigens like tetanus toxin (22, 23). Our results show that (1) Dsg3-specific B cells can be detected at low frequencies in peripheral blood of pemphigus patients, (2) Dsg3-specific memory B cells were significantly increased especially in remitting patients receiving minimal therapy, and (3) isolated Dsg3-specific memory B cells from a PV patient secreted anti-Dsg3 IgG after *in vitro* stimulation. Thus, B cell monitoring with Dsg3-AF647 provides a novel and highly specific tool to investigate the persistence and distribution of autoreactive B cells in PV during the disease course.

## Results

### AF647-Labeled Dsg3 Detects Dsg3-Specific B Cell Clones

**Figure 1:**
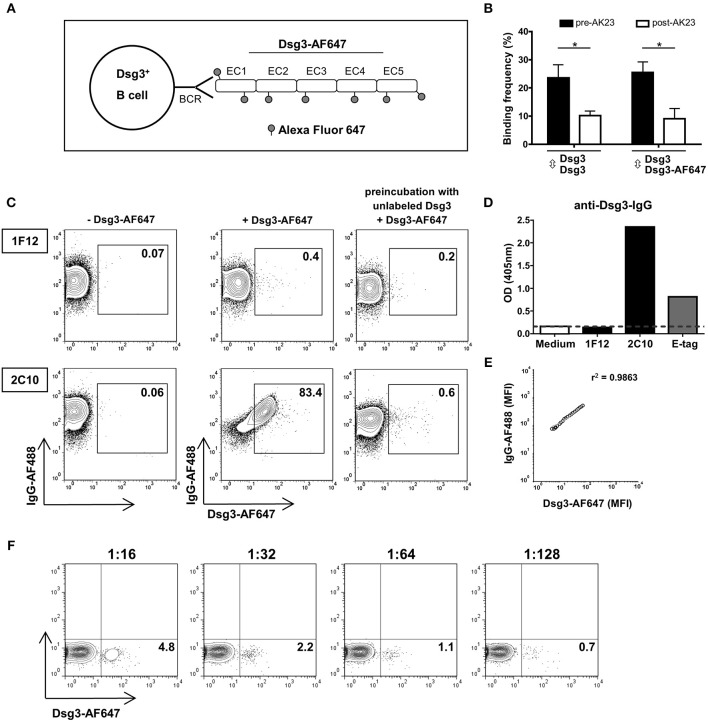
Detection of Dsg3-specific B cell hybridoma (BCH) using fluorescently labeled Dsg3. **(A)** Schematic drawing: the recombinant human extracellular domain (EC1-EC5) of Dsg3 was labeled with the fluorescent dye Alexa Fluor 647 (Dsg3-AF647) and was used for staining of Dsg3-specifc B cell receptors (BCR). **(B)** Binding of Dsg3-AF647 to Dsg3 ± addition of the monoclonal Dsg3-specific antibody AK23 was evaluated with atomic force microscopy. Cumulative data from 3 individual measurements with five replicates for each condition are presented as mean + SD. Statistical analysis was performed by multiple *t*-tests followed by Šidák correction. Differences between groups were considered statistically significant at *p*-values of <0.05 indicated as ^*^. **(C)** Binding efficacy of Dsg3-AF647 to Dsg3-specific BCR was determined by staining of a Dsg3-specific BCH (2C10) and an unrelated BCH (1F12) together with anti-IgG antibody. Binding of Dsg3-AF647 was blocked by preincubation with unlabeled Dsg3. FACS plots shown are representative of three individual experiments. **(D)** Specificity of monoclonal BCH cells for Dsg3 was tested by ELISA. Anti-E-Tag served as positive and culture medium as negative control. **(E)** Correlation of mean fluorescence intensity (MFI) of Dsg3-AF647 with surface IgG. **(F)** Titration of Dsg3-specific BCH (2C10) cells in unrelated 1F12 cells in a calculated ratio of 1:16 (6.25%), 1:32 (3.13%), 1:64 (1.56%), and 1:128 (0.78%) representative of three individual experiments.

In this study we aimed at detecting Dsg3-specific B cells by flow cytometry using fluorescently labeled recombinant Dsg3-AF647 for staining of Dsg3-specific B cell receptors (BCR) as schematically shown in Figure 1A. The fluorescence labeling of recombinant Dsg3 did not functionally impair the interactions between Dsg3-AF647 and Dsg3 compared to homophilic binding of recombinant unlabeled human Dsg3 protein as determined by atomic force microscopy (AFM; Figure 1B). Furthermore, binding of Dsg3-AF647 to Dsg3 was reduced to the same extent compared to unlabeled Dsg3 after adding the monoclonal Dsg3-specific antibody AK23 (24) demonstrating the specificity of this interaction (Figure 1B). To test whether Dsg3-AF647 is capable of binding to Dsg3-specific B cells, the specificity of Dsg3-AF647 staining was evaluated by means of the monoclonal mouse B cell hybridoma (BCH) clone 2C10 producing anti-Dsg3 IgG together with the non-Dsg3-specific BCH clone 1F12 (Figure 1C). Production of anti-Dsg3 IgG by 2C10 was confirmed by ELISA with human Dsg3 protein (Figure 1D). Both BCH clones expressed surface IgG indicating the presence of a functional BCR (Figure 1C). However, only IgG^+^ cells from clone 2C10 showed a strong positive signal upon incubation with Dsg3-AF647 while no specific staining was observed with the control clone 1F12. Binding of Dsg3-AF647 to clone 2C10 was almost completely blocked by preincubation with unlabeled Dsg3 protein (Figure 1C). Of note, the mean fluorescence intensity of Dsg3-AF647 strongly correlated with surface IgG in clone 2C10 (Figure 1E) suggesting a BCR-dependent binding of Dsg3-AF647 to 2C10. In addition, fluorescently labeled human collagen VII (ColVII; noncollagenous domain 1, NC1 (1)) protein which is structurally not related to Dsg3 served as negative control and showed no specific staining in neither of the two BCH cell clones (Supplementary Figure 1B). Finally, we analyzed the sensitivity of Dsg3-AF647 staining by titrating the Dsg3-reactive clone 2C10 with bulk 1F12 cells followed by incubation with Dsg3-AF647 since in peripheral blood of PV patients Dsg3-specific B cells are expected at a low frequency. Our results revealed that Dsg3-specific B cells could be detected within a pool of non-Dsg3-reactive BCH at frequencies of even <1% (Figure 1F).

### Dsg3-Specific B Cells Mainly Appear in the Memory B Cell Pool in PV Patients

**Figure 2:**
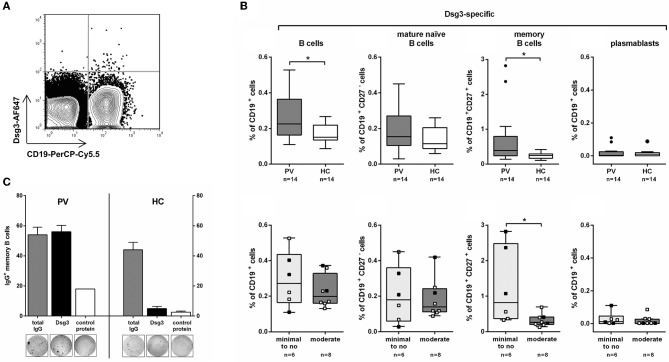
Dsg3-specific memory B cells are increased in remitting PV patients on minimal therapy. **(A)** Representative FACS plot showing staining for CD19^+^Dsg3-AF647^+^ B cells in one PV patient. **(B)** On the upper row: Dsg3-specific B cell populations (total CD19^+^, CD19^+^CD27^−^ mature naïve, CD19^+^CD27^+^ memory, and CD19^+^CD27^hi^CD38^hi^ plasmablasts) were analyzed in PV patients (*n* = 14) and healthy controls (HC; *n* = 14). On the lower row: PV patients were further subdivided based on their systemic treatment into *minimal to no* (*n* = 6) and *moderate* therapy (*n* = 8) showing highest numbers of Dsg3-specific CD19^+^CD27^+^ memory B cells on minimal therapy. ■: after Rituximab treatment; □: no Rituximab treatment. **(C)** CD19^+^CD27^+^Dsg3-AF647^+^ cells were isolated from peripheral blood of a PV patient and a healthy control by FACS sorting and stimulated with R848 and IL-2 to induce plasma cell differentiation. Dsg3-IgG-secreting and total IgG-secreting B cells were subsequently detected using ELISpot by seeding 250 cells per well on ELISpot plates coated either with recombinant human Dsg3 or human collagen VII as control protein. Statistical analysis was performed with two-tailed nonparametric Mann-Whitney-U-Test. Differences between groups were considered statistically significant at *p*-values of <0.05 indicated as ^*^.

Analysis of human Dsg3-specific B cell subpopulations (total CD19^+^ B cells, CD19^+^CD27^−^ B cells, CD19^+^CD27^+^ memory B cells, and CD19^+^CD27^hi^CD38^hi^ plasmablasts) was performed in peripheral blood of 14 clinically well-defined PV patients in either complete or partial clinical remission, or with relapsing disease (Supplementary Tables 1, 2). Individuals with no detectable anti-Dsg3 IgG auto-ab (Supplementary Figure 2) served as healthy control (HC) to determine any potential non-specific staining of Dsg3-AF647 in peripheral blood cells. Since a minor background staining with both Dsg3-AF647 and fluorescently labeled ColVII could also be observed in peripheral blood mononuclear cells of HC, only B cells with a high Dsg3-AF647 signal were considered to be Dsg3-specific (Supplementary Figure 3 for gating scheme). Specificity of Dsg3-AF647 staining in human peripheral blood was also indicated by a high Dsg3-AF647 signal that was only observed in CD19^+^ B cells but not in the CD19^−^ population (Figure 2A).

Dsg3-specific B cells were present in PV patients (0.11–0.53% of CD19^+^ B cells) at higher frequencies compared to HC (0.09–0.22% of CD19^+^ B cells; Figure 2B). The increase in Dsg3^+^ total CD19^+^ B cells was presumably due to the significantly higher number of Dsg3-specific CD19^+^CD27^+^ memory B cells in PV patients. On the other hand, the frequency of Dsg3-specific CD19^+^CD27^−^ B cells (including mature naïve and CD27^−^ memory B cells) did not differ between PV patients and HC (*p* = 0.3; see Supplementary Figure 3) for representative FACS plots). Dsg3-specific CD19^+^CD27^hi^CD38^hi^ plasmablasts were only scarcely detected in peripheral blood of PV patients and HC, respectively. To determine whether Dsg3-specific memory B cells in patients with PV are capable of producing anti-Dsg3 IgG, we sorted CD19^+^CD27^+^Dsg3-AF647^+^ cells from a PV patient in clinical remission with persistent anti-Dsg3 IgG titers. We could demonstrate that *in vitro* stimulation of sorted CD19^+^CD27^+^Dsg3-AF647^+^ cells with toll-like receptor agonist R848 and interleukin-2 (IL-2) leads to plasma cell differentiation (according to Pinna et al. (25)) and that Dsg3-specific IgG-producing B cells were detected to a similar extent as IgG secreting cells in PV by ELISpot assay (Figure 2C). In contrast, although sorted Dsg3-AF647^+^ memory B cells from HC showed a marked IgG production, the majority of these cells did not possess the capacity to produce anti-Dsg3 IgG (Figure 2C).

### Remitting PV Patients Show the Highest Number of Dsg3-Specific Memory B Cells

As some of the PV patients received systemic treatment (systemic corticosteroids, other adjuvant immunosuppressives, B cell depletion) they were subdivided into two groups with *minimal to no* therapy (including 6 remitting patients) or *moderate* therapy (including 8 patients with relapsing disease or partial remission; Supplementary Table 1). Interestingly, Dsg3-specific memory B cells were only increased in remitting PV patients receiving *minimal* therapy. On *moderate* treatment Dsg3-specific B cells were hardly detected similar to HC (Figure 2B). Here we could show that systemic immunosuppressive treatment seems to have an impact on the frequency of circulating autoreactive B cells in PV. Of note, in the group of remitting PV patients the highest numbers of Dsg3-specific memory B cells exceeding the levels observed in HC were particularly detected in patients who underwent previous B cell depletion (Patient 4, 8, 13 in Supplementary Table 1) suggesting a reconstitution of autoreactive memory B cells upon anti-CD20 antibody treatment (Supplementary Figure 4).

## Discussion

In this study, we demonstrate by use of fluorescently labeled Dsg3 protein the detection of low-frequent Dsg3-specific autoreactive B cells in peripheral blood of PV patients. Flow cytometric analysis of B cell subpopulations with Dsg3-AF647 revealed significantly higher numbers of Dsg3-specific B cells in PV patients compared to HC, particularly within the memory B cell compartment.

Our present findings are in line with Nishifuji et al. (26) who detected circulating Dsg3-specific memory B cells in PV patients but not in HC using ELISpot analysis with a general low frequency (6.3–84.0 cells per 10^5^ PBMC). This is similar to our study where we found low-frequent Dsg3-specific B cells in PV patients (0.11–0.53% of CD19^+^ B cells) even though the acquired percentages can only be compared to a limited extent due to the different assays used. In the work by Nishifuji et al., Dsg3-specific B cells spontaneously producing anti-Dsg3 IgG were exclusively observed in patients with the highest disease activity while Dsg3-specific memory B cells could only be detected after *in vitro* stimulation in a group of nine out of 14 patients (26). Of note, this group included patients with low or no disease activity pointing toward a persistence of Dsg3-specific memory B cells in patients in complete or partial remission which we could also observe in our study as Dsg3-specific CD19^+^CD27^+^ memory B cells were significantly increased in PV patients compared to HC. Since the generation of CD27^+^ memory B cells is thought to be induced in germinal centers and requires collaboration with T follicular helper (Tfh) cells (27), the presence of autoreactive memory B cells in PV patients points toward a defective Tfh cell tolerance checkpoint within the germinal center response in pemphigus as recently suggested by our group (28).

Most interestingly, the highest numbers of Dsg3-specific memory B cells were observed in 3 PV patients ~2 years after treatment with Rituximab (22–27 months; patient 4, 8, and 13; Supplementary Table 1), while increasing Dsg3-specific memory B cells could not be observed in a PV patient (patient 6) who experienced a relapse 35 months after rituximab (Supplementary Figure 4). Since disease relapses after rituximab treatment do often occur at a time point starting from 1 year after treatment (18, 19) this observation may point toward an ongoing reconstitution of autoreactive memory B cells. Those memory B cells would initially not produce autoantibodies however, upon immune activation with Dsg3 and other trigger factors, they possess the capability to rapidly differentiate into autoantibody-secreting plasma cells, hence providing the base for a potential disease relapse.

However, Dsg3-specific plasmablasts, representing antibody-secreting cells, were only scarcely detected in our study despite high anti-Dsg3 IgG titers in individual patients. This might be explained by their reduced expression of surface immunoglobulin which limits the capability for detecting these cells using fluorescently labeled Dsg3 (29). Furthermore, due to the restriction to peripheral blood for analysis of Dsg3-specific B cells we were not able to identify autoreactive plasma cells residing within the niches of lymphoid tissues or bone marrow that might account for the continuous secretion of autoreactive autoantibodies in PV. Nonetheless, clonal analysis of autoreactive B cells in PV showed that Dsg3-specific B cells in peripheral blood can persist in PV patients for many years during active disease, clinical remission or even after B cell-depleting therapy indicating the suitability of peripheral blood for monitoring autoreactive B cell responses (20). Whether these B cells are newly generated or persistent cells that were not completely removed by immunosuppressive therapy is still under investigation. Recent results showed that autoreactive B cells could persist in patients with PV but also in patients with lupus erythematosus (SLE) and to a much lower frequency in healthy individuals (30, 31).

In general, anti-Dsg3 IgG is found at a very low prevalence in healthy individuals (32), thus the low frequency of Dsg3-specific B cells in HC is suggestive of IgM^+^ B cells producing non-pathogenic, potentially cross-reactive natural IgM antibodies (33, 34). However, the observed signal for Dsg3-AF647 in HC may also be in part due to unspecific binding of the fluorescently labeled protein to B cells. Hence, inclusion of unspecific staining in samples from patients and controls was minimized to the best possible extent as only B cells with a very high Dsg3-AF647 signal were considered to be Dsg3-specific (Figure 2A).

To summarize, the present identification of peripheral blood Dsg3-reactive B cells in peripheral blood of PV patients provides further insights into the autoimmune B cell repertoire in PV. Monitoring of Dsg3-specific peripheral B cells with a special focus on Dsg3-specific CD19^+^CD27^+^ memory B cells might be a predictive tool to determine the effectiveness of therapeutic interventions in patients with PV. Particularly as recent studies suggest that clinical remission in PV upon therapy is associated with an increase of IL-10-secreting B cells downregulating B cell activation (18, 35), the extended flow cytometric analysis of Dsg3-specific IL-10-producing B cells in PV patients at different disease stages can provide further insights into the individual progression of disease.

## Materials and Methods

### Human Subjects

Peripheral blood from 14 PV patients (Supplementary Table 1) as well as 14 HC with no signs of autoimmune skin inflammation and no serum anti-Dsg3 IgG (Supplementary Figure 2) was drawn into citrate-phospate-dextrose-adenine (CPDA) anticoagulant. Each study participant gave written informed consent before inclusion in the study, which was approved by the Ethics Committee of the Medical Faculty of the Philipps-Universität, Marburg (Az. 20/14). The study was conducted in accordance with the Declaration of Helsinski.

### Fluorescent Labeling of Recombinant Human Dsg3

Recombinant human Dsg3 (extracellular domain, aa 1-566), produced in the baculovirus expression system (36, 37), was fluorescently labeled using the AlexaFluor647 Labeling kit (Thermo Fisher Scientific, Schwerte, Germany) according to the manufacturers' protocol.

### Detection of Dsg3-Specific B Cells

Peripheral blood mononuclear cells (PBMC) were isolated from peripheral blood using Lymphocyte Separation Medium (Capricorn, Ebsdorfergrund, Germany). Mouse BCH clones were cultured in RPMI-1640 supplemented with 100 U/ml penicillin, 100 μg/ml streptomycin, and 2 mM L-glutamine (all Capricorn) and 10% FCS (Merck Millipore, Berlin, Germany). PBMC were washed twice with PBS + 1% FCS and 1 ×10^6^ cells per sample were subsequently stained with Dsg3-AF647 together with mouse anti-human CD19-PerCP-Cy5.5 (HIB19), mouse anti-human CD27-PE (M-T271), mouse anti-human CD38-FITC (HIT2) and the respective isotype controls (all BD Biosciences, Heidelberg, Germany) or with goat anti-mouse IgG-AF488 (A-11029; Thermo Fisher Scientific, Waltham, MA, USA) for mouse BCH clones. After incubation for 20 min at 4°C cells were washed twice with PBS + 1% FCS and a minimum of 2.5 ×10^5^ PBMC or 0.5 ×10^5^ BCH per sample were acquired on a FACS Calibur (BD Biosciences). In a subset of experiments, sorting of cells was performed using FACS Aria III (BD Biosciences). Data analysis was performed using FlowJo 7.6 (TreeStar Inc., Ashland, USA).

### ELISpot Assay

Dsg3-specific B cells (CD19^+^CD27^+^Dsg3-AF647^+^) were isolated from PBMC by FACS sorting. 2 ×10^3^ cells were seeded in 96-well U-bottom microplates and cultured in RPMI-1640 +100 U/ml penicillin, +100 μg/ml streptomycin, +2 mM L-glutamine +10% FCS together with 2.5 μg/ml R848 (Mabtech AB, Nacka Strand, Sweden), and 1,000 U/ml human recombinant IL-2 (Roche, Mannheim, Germany) as previously described (25). After 5 days, cells were seeded at 250 cells/well in duplicates on an ELISpot plate coated with recombinant human Dsg3 or collagen VII and incubated for 20 h at 37°C in a humidified atmosphere containing 5% CO_2_. Detection of Dsg3-specific IgG^+^ spots was performed according to the manufacturer's protocol (Mabtech AB) and spots were counted automatically with an ELISpot plate reader (A.EL.VIS, Hanover, Germany).

### Atomic Force Microscopy

Cell-free AFM measurements were performed on a NanoWizard 3 AFM (JPK-Instruments, Berlin, Germany) as previously described (38) (see Supplementary Material for detailed description).

### Detection of Anti-Dsg IgG

The presence of IgG auto-ab against Dsg1 or Dsg3 in blood of PV patients and HC was evaluated by anti-Dsg1- and anti-Dsg3-ELISA (Euroimmun, Lübeck, Germany) according to the manufacturer's protocol.

### Statistical Analysis

Statistical analysis was performed using GraphPad Prism 6.02 (GraphPad Software Inc., La Jolla, USA). Cumulative data are displayed as box plots with median. For group comparisons two-tailed nonparametric Mann-Whitney-U-Test was applied. Data from AFM experiments were statistically evaluated by multiple *t*-tests followed by Šidák correction. Differences between the groups were considered as statistically significant at *p* values <0.05.

## Data Availability

All datasets generated for this study are included in the manuscript and/or the Supplementary Files.

## Ethics Statement

Each study participant gave written informed consent before inclusion in the study, which was approved by the Ethics Committee of the Medical Faculty of the Philipps-Universität, Marburg (Az. 20/14). The study was conducted in accordance with the Declaration of Helsinski.

## Author Contributions

RP performed the experiments, analyzed the data, and wrote the manuscript. EW performed and analyzed the AFM experiments. TS, JW, MH, and CM participated to the study design, contributed to the manuscript, and supervised the study. RE conceived the study, recruited pemphigus patients, and revised the manuscript.

### Conflict of Interest Statement

The authors declare that the research was conducted in the absence of any commercial or financial relationships that could be construed as a potential conflict of interest.

## Abbreviations

AF647: Alexa Fluor 647
AFM: atomic force microscopy
auto-ab: autoantibodies
BCH: B cell hybridoma
BCR: B cell receptor
COLVII: type VII collagen
Dsg: desmoglein
HC: healthy controls
PV: pemphigus vulgaris.
