# Supplementary material for: Identification of Autoreactive B Cell Subpopulations in Peripheral Blood of Autoimmune Patients With Pemphigus Vulgaris
Source: Front Immunol. 2019 Jun 14;10:1375. doi: 10.3389/fimmu.2019.01375 (PMC6587433; doi:10.3389/fimmu.2019.01375)
Supplement: Supplementary file 1 [file Data_Sheet_1.docx]

Supplementary Material

**Identification of Autoreactive B cell Subpopulations in Peripheral Blood of Autoimmune Patients with Pemphigus vulgaris**

**Robert Pollmann, Elias Walter, Thomas Schmidt, Jens Waschke, Michael Hertl, Christian Möbs, Rüdiger Eming^*^**

*** Correspondence:** Rüdiger Eming: [eming@med.uni-marburg.de](mailto:eming@med.uni-marburg.de)

# Supplementary Figures

**
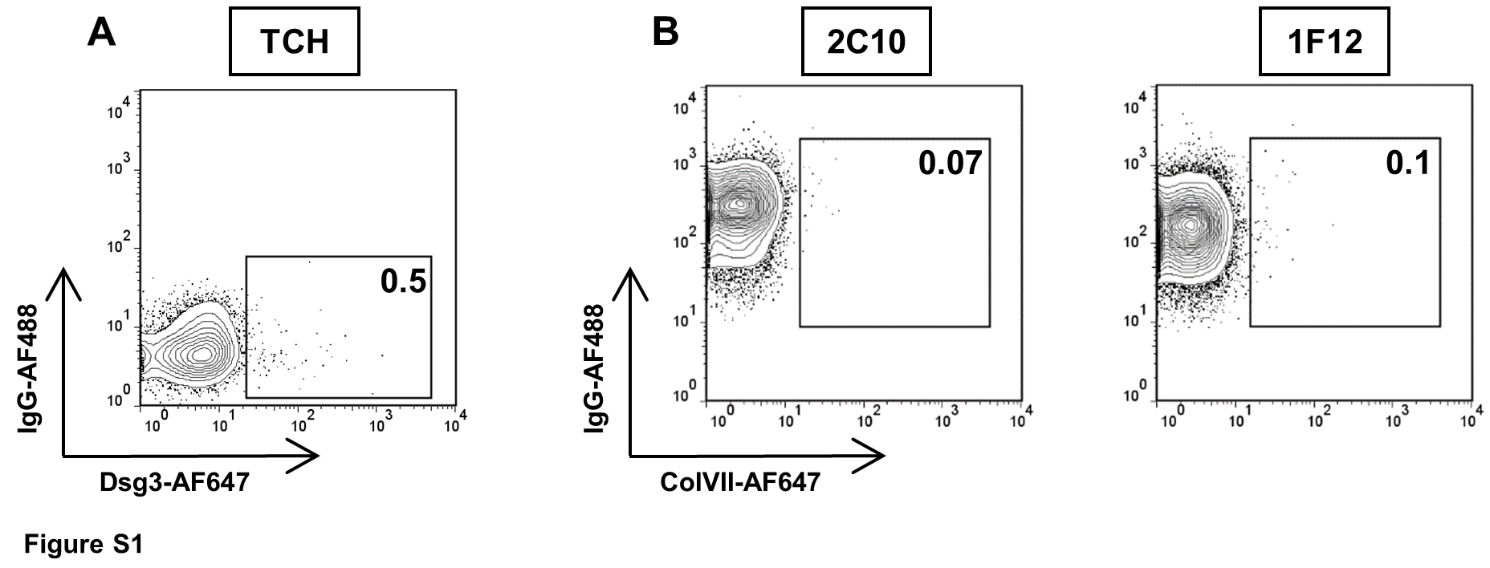
**

**Supplementary Figure 1.** **(A)** Staining of a IgG^–^ T cell hybridoma (TCH) clone with fluorescently labeled Dsg3. **(B)** Staining of IgG^+^ B cell hybridoma (BCH) clones specific for Dsg3 (2C10) or with unknown specificity (1F12) with fluorescently labeled type VII collagen protein (ColVII).


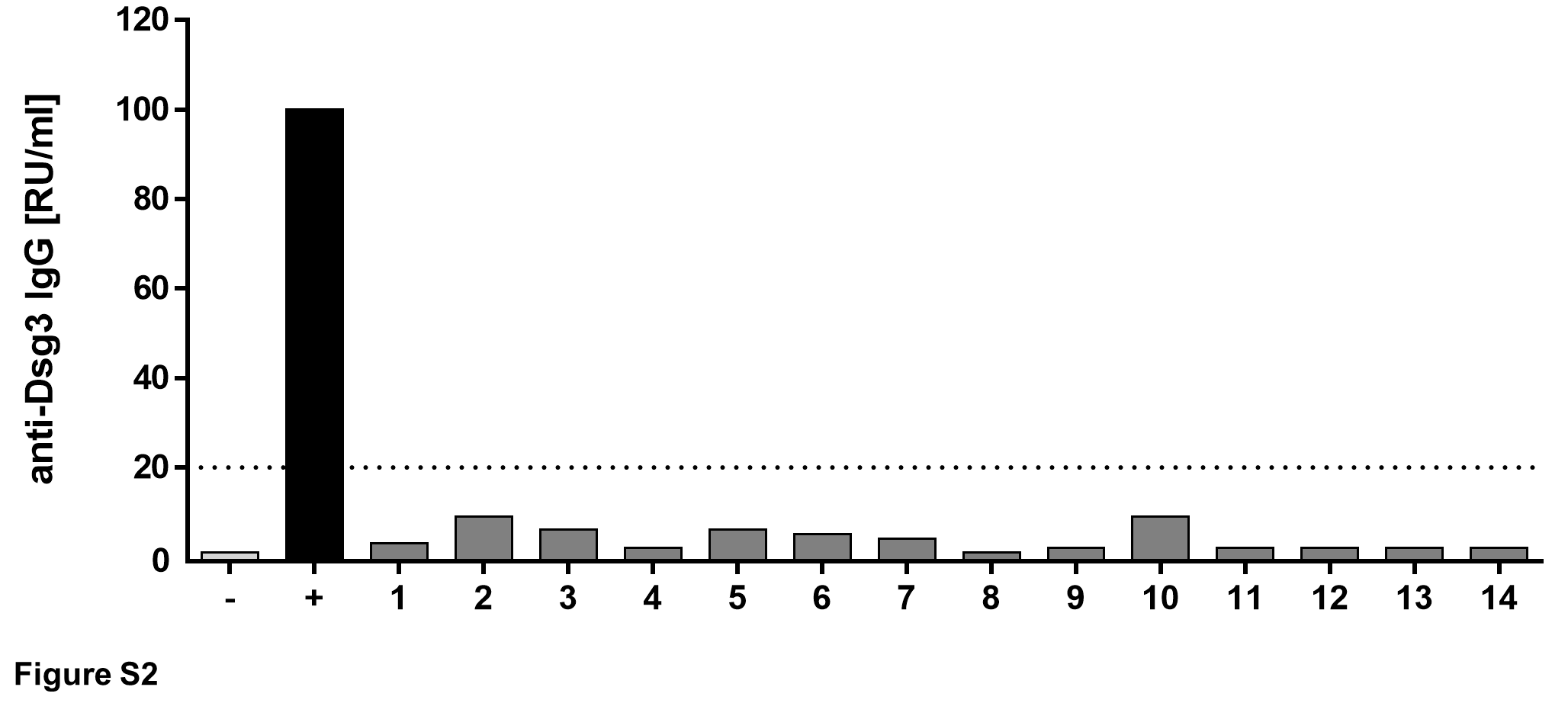


**Supplementary Figure 2.** Anti-Dsg3-ELISA with plasma of healthy controls (n=14). Values are shown as relative units (RU) per ml; cut-off value for positive result= 20 RU/ml (dotted line).
**–**: negative control, **+**: positive control.


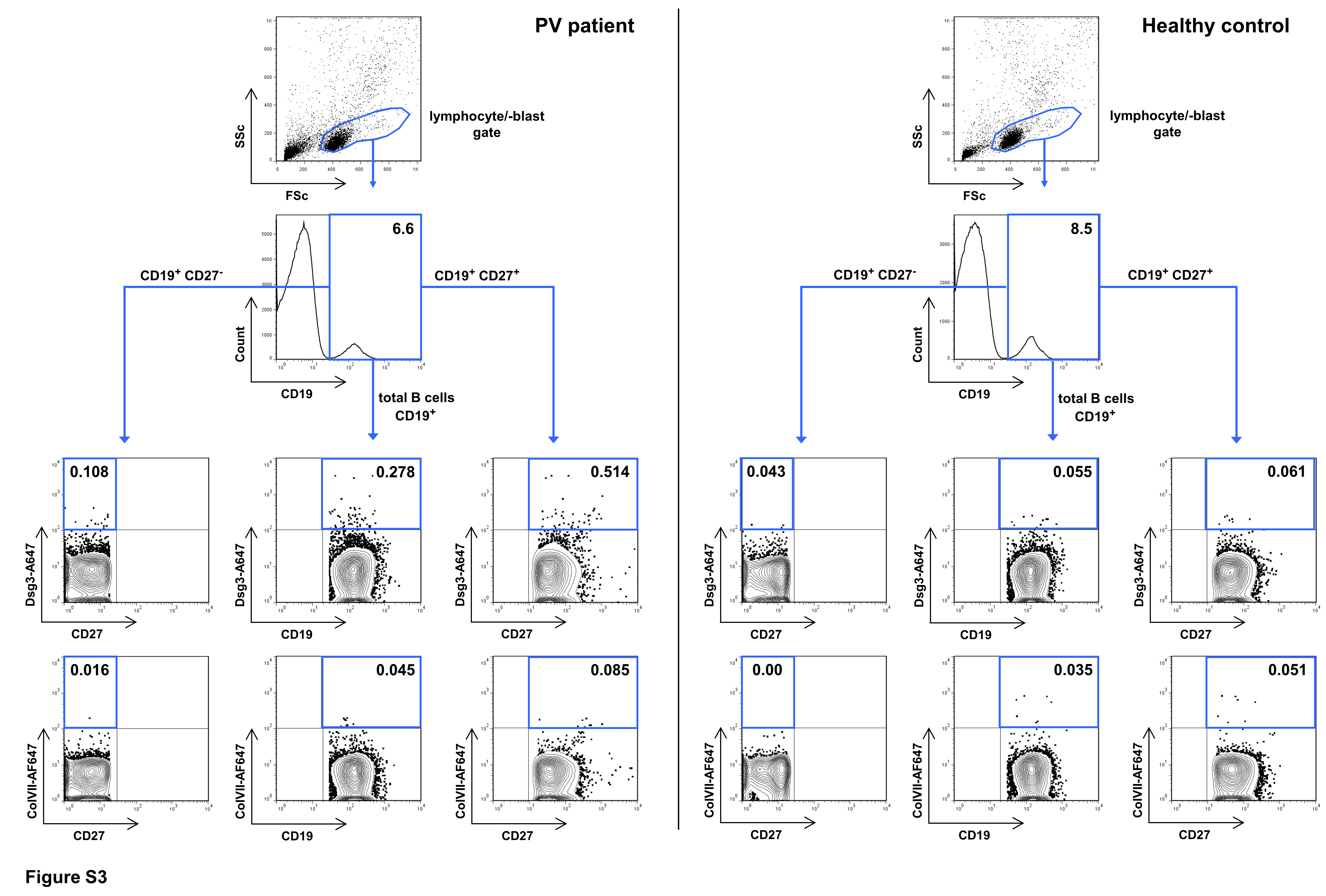


**Supplementary Figure 3.** Gating strategy for analysis of Dsg3-specific B cell subpopulations in human peripheral blood. Lymphocytes and lymphoblasts were gated on size and granularity. B cells were gated on CD19 expression and Dsg3-specific B cells were subdivided into total CD19^+^ B cells, CD19^+^CD27^–^ and CD19^+^CD27^+^ (memory) B cells. Collagen VII was used as non-specific control. Shown are representative results from one PV patient and one healthy control.


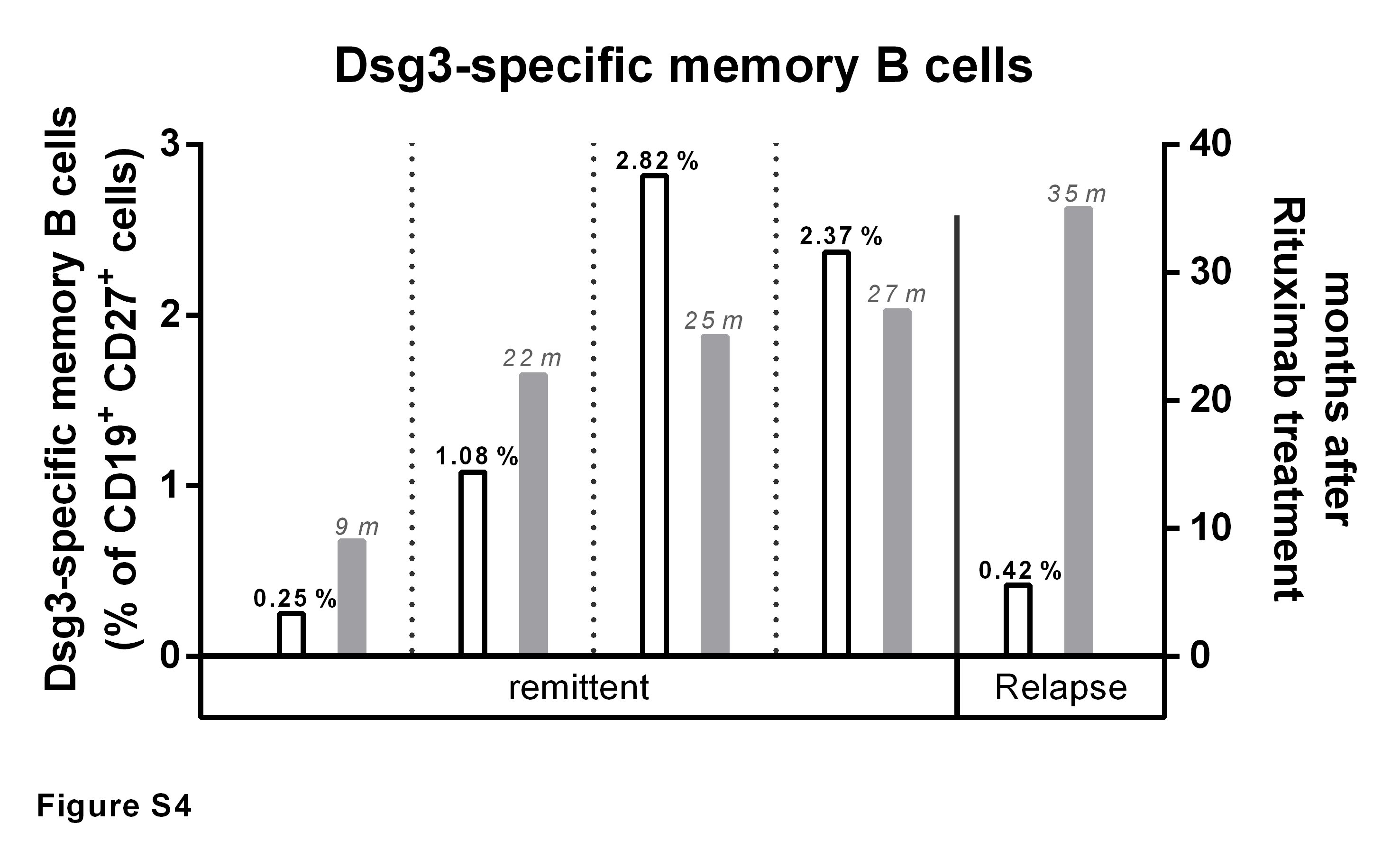
**Supplementary Figure 4.** Frequencies of Dsg3-specific memory B cells in the five rituximab-treated PV patients included in this study (see Table S1) with regard to disease status and months (m) after end of Rituximab treatment. The highest frequencies of autoreactive memory B cells can be observed in remitting PV patients after ~2 years (22-27 months) after treatment.

# Supplementary Tables

**Supplementary Table 1:** Disease status, treatment and auto-ab profile of patients with pemphigus vulgaris.

| Patient | Status^1^ | Therapy | Medication^2^ | Clinical phenotype | | Rituximab^3^ | Auto-ab (IgG)^4^ | |
| --- | --- | --- | --- | --- | --- | --- | --- | --- |
|  |  |  |  | Skin | Mucosa |  | Dsg3 | Dsg1 |
| 1 | Partial Remission  (on therapy) | minimal | 10mg Pred^5^ | - | oral | - | 72 | - |
| 2 | Complete Remission  (off therapy) | no | None | - | - | - | 176 | - |
| 3 | Partial Remission  (on therapy) | moderate | 20mg Pred  2g MMF^6^ | - | oral | - | 72 | - |
| 4 | Complete Remission  (off therapy) | no | None | - | - | 25 | - | - |
| 5 | Relapse | moderate | 20mg Pred  2g MMF | head | oral, genital | - | 1445 | 122 |
| 6 | Partial Remission  (on therapy) | moderate | 20mg Pred  150mg Aza^7^ | head, trunk, legs | - | 9 | 121 | 182 |
| 7 | Partial Remission  (on therapy) | moderate | 15mg Pred  50mg Dapson | - | oral | - | 529 | - |
| 8 | Partial Remission  (on therapy) | minimal | 5mg Pred | - | oral | 22 | 281 | 94 |
| 9 | Complete Remission  (on therapy) | minimal | 5mg Pred | - | - | - | 94 | - |
| 10 | Relapse | moderate | 40mg Pred | trunk, arms, legs | oral | 35 | 75 | 1582 |
| 11 | Relapse | moderate | 20mg Pred  150mg Aza | head | - | - | 5619 | - |
| 12 | Partial Remission  (on therapy) | moderate | 15mg Pred  1g MMF | trunk, arms, legs | - | - | - | - |
| 13 | Partial Remission  (off therapy) | no | None | - | oral | 27 | 606 | - |
| 14 | Relapse | moderate | 30mg Pred | trunk, head, feet | oral | - | 330 | 145 |

^1^ as defined by Murrell et al. (1)

^2^ daily medication at time of study

^3^ months after Rituximab treatment

^4^ as determined by ELISA with recombinant Dsg1 or Dsg3 in RU/mL. The cut-off value is 20 RU/mL

^5^ prednisolone

^6^ mycophenolate mofetil

^7^ azathioprine

| **PV** | B cells | | |  | Dsg3-specific B cells | | | | |  |  | **HC** | B cells | | |  | Dsg3-specific B cells | | | | |  |  |
| --- | --- | --- | --- | --- | --- | --- | --- | --- | --- | --- | --- | --- | --- | --- | --- | --- | --- | --- | --- | --- | --- | --- | --- |
|  | all | naïve | memory |  | all | naïve | | memory | |  |  |  | all | naïve | memory |  | all | naïve | | memory | |  |  |
|  | (a) | (b) | (b) |  | (b) | (b) | (c) | (b) | (d) |  |  |  | (a) | (b) | (b) |  | (b) | (b) | (c) | (b) | (d) |  |  |
| 1. | 7.72 | 51.33 | 48.67 |  | 0.22 | 0.04 | 0.07 | 0.19 | 0.38 |  |  | 1. | 5.48 | 85.07 | 14.84 |  | 0.15 | 0.10 | 0.11 | 0.06 | 0.42 |  |  |
| 2. | 5.27 | 38.90 | 59.66 |  | 0.53 | 0.17 | 0.45 | 0.36 | 0.57 |  |  | 2. | 10.30 | 83.28 | 16.58 |  | 0.09 | 0.07 | 0.08 | 0.02 | 0.11 |  |  |
| 3. | 5.46 | 60.89 | 39.02 |  | 0.16 | 0.07 | 0.12 | 0.09 | 0.41 |  |  | 3. | 12.70 | 64.10 | 35.75 |  | 0.15 | 0.05 | 0.07 | 0.10 | 0.29 |  |  |
| 4. | 13.30 | 95.52 | 4.411 |  | 0.32 | 0.20 | 0.21 | 0.12 | 2.82 |  |  | 4. | 8.47 | 34.74 | 64.92 |  | 0.06 | 0.03 | 0.04 | 0.09 | 0.06 |  |  |
| 5. | 5.90 | 33.32 | 65.91 |  | 0.13 | 0.04 | 0.11 | 0.09 | 0.14 |  |  | 5. | 9.19 | 55.22 | 44.91 |  | 0.13 | 0.05 | 0.09 | 0.08 | 0.17 |  |  |
| 6. | 0.75 | 73.86 | 24.20 |  | 0.37 | 0.31 | 0.42 | 0.06 | 0.25 |  |  | 6. | 14.60 | 71.80 | 28.15 |  | 0.22 | 0.14 | 0.19 | 0.08 | 0.30 |  |  |
| 7. | 5.61 | 62.26 | 37.17 |  | 0.23 | 0.15 | 0.25 | 0.08 | 0.20 |  |  | 7. | 6.95 | 57.42 | 41.89 |  | 0.22 | 0.15 | 0.26 | 0.07 | 0.16 |  |  |
| 8. | 3.97 | 94.42 | 5.178 |  | 0.40 | 0.30 | 0.33 | 0.10 | 1.08 |  |  | 8. | 6.64 | 76.31 | 23.43 |  | 0.19 | 0.11 | 0.14 | 0.09 | 0.37 |  |  |
| 9. | 8.24 | 81.60 | 18.37 |  | 0.18 | 0.12 | 0.15 | 0.06 | 0.34 |  |  | 9. | 11.10 | 74.82 | 24.20 |  | 0.14 | 0.08 | 0.11 | 0.06 | 0.25 |  |  |
| 10. | 15.90 | 95.76 | 4.24 |  | 0.23 | 0.21 | 0.22 | 0.02 | 0.42 |  |  | 10. | 9.90 | 61.30 | 38.61 |  | 0.17 | 0.08 | 0.13 | 0.09 | 0.25 |  |  |
| 11. | 6.57 | 63.60 | 35.70 |  | 0.28 | 0.07 | 0.11 | 0.29 | 0.52 |  |  | 11. | 11.60 | 60.20 | 39.70 |  | 0.24 | 0.15 | 0.25 | 0.10 | 0.24 |  |  |
| 12. | 11.00 | 61.90 | 37.89 |  | 0.17 | 0.06 | 0.09 | 0.11 | 0.28 |  |  | 12. | 9.23 | 72.40 | 27.01 |  | 0.27 | 0.19 | 0.26 | 0.08 | 0.29 |  |  |
| 13. | 18.80 | 97.46 | 2.50 |  | 0.11 | 0.03 | 0.03 | 0.08 | 2.37 |  |  | 13. | 9.39 | 74.30 | 25.24 |  | 0.14 | 0.09 | 0.12 | 0.05 | 0.19 |  |  |
| 14. | 5.15 | 84.06 | 15.94 |  | 0.17 | 0.14 | 0.16 | 0.04 | 0.23 |  |  | 14. | 8.89 | 63.50 | 36.34 |  | 0.13 | 0.04 | 0.06 | 0.09 | 0.26 |  |  |
|  |  |  |  |  |  |  |  |  |  |  |  |  |  |  |  |  |  |  |  |  |  |  |  |
| **PV** |  |  |  |  |  |  |  |  |  |  |  | **HC** |  |  |  |  |  |  |  |  |  |  |  |
| *Max.* | 18.80 | 97.46 | 65.91 |  | 0.53 | 0.31 | 0.45 | 0.36 | 2.82 |  |  | *Max.* | 14.60 | 85.07 | 64.92 |  | 0.22 | 0.15 | 0.26 | 0.10 | 0.42 |  |  |
| *Median* | 6.24 | 68.73 | 29.95 |  | 0.23 | 0.14 | 0.15 | 0.09 | 0.40 |  |  | *Median* | 9.31 | 67.95 | 31.95 |  | 0.15 | 0.08 | 0.11 | 0.08 | 0.21 |  |  |
| *Min.* | 0.75 | 33.32 | 2.50 |  | 0.11 | 0.03 | 0.03 | 0.02 | 0.14 |  |  | *Min.* | 5.48 | 34.74 | 14.84 |  | 0.09 | 0.04 | 0.07 | 0.02 | 0.11 |  |  |

**Supplementary Table 2:** Frequencies of B cell subpopulations in patients with pemphigus vulgaris (PV) and healthy controls (HC).

(a): % CD19^+^ cells of gated lymphocytes / -blasts

(b): % of CD19^+^ B cells

(c): % of CD19^+^CD27^–^ B cells

(d) % of CD19^+^CD27^+^ memory B cells

# Supplementary Methods

**Atomic Force Microscopy**

Mica-sheets where freshly cleaved and coated together with Si_3_N_4_ MLCT Cantilevers (Bruker, Mannheim, Germany) with heterobifunctional acetal-polyethylenglycol linker (PEG, Hermann Gruber Lab, Institute of Biophysics, Linz, Austria). The PEG-linker in turn was linked to either recombinant human Dsg3 or labeled recombinant human Dsg3-AF647 functionalizing the mica-sheet and cantilever. The spring constant of the D-cantilever was determined utilizing the thermal noise calibration method (2). Force maps were generated in force mapping mode in a 10x10µm area with one measurement per µm. Five maps were generated with probing settings set to a pulling speed of 1µm/s and a pulling distance of 0.3µm with a resting time of 0.1s in HBSS buffer. Cantilever deflections were converted to force distant curves to determine the presence of binding events. After application of AK23 (Biozol, Eching, Germany) in a concentration of 75µg/ml for 1 hour the same area was measured again. Eventually the binding frequency was determined by evaluating both datasets for unbinding events.

# Supplementary References

1 **Murrell, D. F., Dick, S., Ahmed, A. R., Amagai, M., Barnadas, M. A., Borradori, L. and Bystryn, J.-C. et al.,** Consensus statement on definitions of disease, end points, and therapeutic response for pemphigus. *J Am Acad Dermatol.* 2008. **58**(6): 1043–1046

2 **Slattery, A. D., Blanch, A. J., Quinton, J. S. and Gibson, C. T.,** Accurate measurement of Atomic Force Microscope cantilever deflection excluding tip-surface contact with application to force calibration. *Ultramicroscopy.* 2013. **131**: 46–55
